# Lenticels are sites of initiation of microcracking and russeting in ‘Apple’ mango

**DOI:** 10.1371/journal.pone.0291129

**Published:** 2023-09-01

**Authors:** Thomas O. Athoo, Andreas Winkler, Willis O. Owino, Moritz Knoche

**Affiliations:** 1 Institute for Horticultural Production Systems, Leibniz-University Hannover, Hannover, Germany; 2 School of Food and Nutritional Sciences (SOFNUS), Jomo Kenyatta University of Agriculture and Technology, Nairobi, Kenya; Universidad Autónoma Agraria Antonio Narro, MEXICO

## Abstract

The mango cultivar ‘Apple’ is an important fruitcrop in Kenya, but it is highly susceptible to russeting. The objective was to establish whether lenticels predispose cv. ‘Apple’ mango to russeting. Fruit mass and surface area increased in a sigmoidal pattern with time. The frequency of lenticels per unit surface area decreased during development. The number of lenticels per fruit was constant. Lenticels were most frequent in the apex region and least common in the cheek and nak (ventral) regions. The cheek region also had lenticels with the largest core areas, whereas the lenticel core areas in the apex region were significantly smaller. Microscopy revealed stomata became covered over with wax deposits at 33 days after full bloom (DAFB). By 78 DAFB, periderm had formed beneath the pore. At 110 and 161 DAFB, cracks had developed and the periderm had extended tangentially and radially. The presence of lenticels increased the strain released upon excision of an epidermal segment, further strain releases occurred subsequently upon isolation of the cuticle and on extraction of the cuticular waxes. The number of lenticels per unit surface area was negatively correlated with the fruit surface area (r^2^ = 0.62 **), but not affected by fruit size. Mango cv. ‘Apple’ had fewer, larger lenticels and more russet, compared with ‘Ngowe’, ‘Kitovu’ or ‘Tommy Atkins’ mango. In cv. ‘Apple’, the lowest lenticel frequency, the largest lenticels and the most russeting occurred at a growing site at the highest altitude, with the highest rainfall and the lowest temperature. Moisture exposure of the fruit surface resulted in enlarged lenticels and more microcracking of the cuticle. Our results establish that russeting in ‘Apple’ mango is initiated at lenticels and is exacerbated if lenticels are exposed to moisture.

## Introduction

Surfaces of young fruit are often stomatous. Stomata regulate gas exchange by changes in their conductance mediated through the opening and closing of the stomatal pore, usually in response to environmental stimuli [1]. In many fruit crops, stomata on the fruit surface later develop into lenticels [1, 2]. In contrast to a stomate, the conductance of a lenticel is not regulated. Anatomically, lenticels represent a periderm comprising a phellogen that produces stacks of cork cells, the phellem [2]. The phellem that fills the core of these lenticels comprises a volume of loosely packed cells [2, 3], thereby facilitating gas exchange [1, 2]. When the epidermis and cuticle are sloughed off later on during fruit development, the phellem forms the new surface. Lenticels then turn reddish/brown as a result of the suberization of their cell walls.

Lenticels increase the fruit skin’s permeance to postharvest water loss [4, 5]. Furthermore, in some fruit crops the lenticels represent sites of preferential infection with pathogens [6] but there is only limited evidence for this last observation in mango [7].

In mango (*Mangifera indica* L.), two important physiological disorders are associated with lenticels—lenticel discoloration and russeting. Lenticel discoloration occurs postharvest and involves the deposition of darkly pigmented phenolics in a distinct zone around the lenticel [8–10]. In contrast, russet develops preharvest and usually covers larger portions of the fruit surface [11]. There is some indication that mechanical stress can be involved in both lenticel discoloration and in russeting [12–14].

The mango cv. ‘Apple’ is grown widely in Kenya [15]. However, it is highly susceptible to russeting. The skin of a russeted fruit is dull brown and rough [11]. Russeted fruit shrivel faster postharvest [11]. An earlier study suggests russeting in cv. ‘Apple’ mango is initiated at lenticels [11]. Furthermore, that russeting is triggered by surface wetness [11, 16]. Unfortunately, little is known about the development of lenticels in mango in general [14], nor for russeting in cv. ‘Apple’ mango in particular. It is not known if or how lenticel development is affected by exposure to surface moisture.

The objective of this study was to identify if and how lenticels predispose cv. ‘Apple’ mango fruit to russeting. The effects of exposure of the fruit to surface moisture on later russeting development were also investigated.

## Materials and methods

### Plant materials

Fruits of the mango cvs. ‘Apple’, ‘Kitovu’, ‘Ngowe’ and ‘Tommy Atkins’ were obtained from a range of commercial orchards in Kenya. From Kakuzi (Murang County, altitude 1327 m) (1°04’S, 37°19’E), Kibwezi (Makueni County, 687 m) (2°20’S, 38°07’E) and Mwala (Machakos County, 1244 m) (1°19’S, 37°26’E). All fruit were grown using integrated pest management programs. Fruit were sampled randomly from trees preselected for uniformity in flowering and tree size and shape. Border trees were avoided. The fruit selected for sampling was free of visible defects and representative in size and color for the population of fruit on the tree. Fruit was processed within 48 hours of sampling.

### Methods

#### Microscopic inspection of the fruit surface

Stomata and lenticels were inspected microscopically. Unless specified otherwise, epidermal segments (ES) were excised from the cheek region of the fruit using a razor blade. The ES were examined under a stereo microscope (MZ10F; Leica Microsystems, Wetzlar, Germany) and photographed (Camera DFC7000T; Leica Microsystems). The magnifications were such that the rectangular windows on the fruit skin selected for measurement ranged from 1.7 × 1.3 mm to 13.9 × 10.5 mm. The lenticel frequency (number of lenticels per unit area), the core area and the pore area (opening) per lenticel were quantified by image analysis (ImageJ 1.53P; National Health Institute, Bethesda, MD, USA). See supplementary **S1 Fig** for an illustration of the core and pore areas of a lenticel.

Stomata were investigated before and after the removal of the epicuticular wax. The epicuticular wax was removed by dipping the fruit in a 1:1 (v:v) chloroform: methanol mixture (CHCl_3_:CH_3_OH) for 15 min. The ES were viewed using a digital microscope (VHX-7100; Keyence corporation, Osaka, Japan). The magnification was 1500× (objective VHX-E500, Keyence).

#### Microscopic inspection of cross-sections

Pieces of the skin and adhering flesh were excised using a razor blade and fixed in a formaldehyde-glutaraldehyde solution [17]. Following rinsing, tissue blocks comprising a portion of the skin and adhering flesh (approx. 5 x 2 x 2 mm) were cut by hand. The blocks were placed inside plastic cassettes (PrintMate biopsy Cassetes; Thermo Fisher Scientific, Kalamazoo, MI, USA), immersed in 70% ethanol and stored overnight at 4°C. The blocks were vacuum infiltrated at 10.8 kPa absolute pressure for 30 min each with aqueous ethanol (70, 80, 90 and 96% v:v) and twice with absolute isopropanol. The blocks were further vacuum infiltrated twice for 40 min with xylene substitute (AppliClear; PanReac Applichem, Muenster, Germany), then once for 40 min in a 1:1 v:v paraffin/xylene substitute mixture. Subsequently, the blocks were vacuum infiltrated in melted paraffin, held at 65°C for 12–14 h (Memmert 100–800; Memmert, Schwabach, Germany) and then embedded in melted paraffin. The embedded blocks were stored at 4°C until use.

Thin sections (10 μm) of embedded tissue were cut with a microtome (Hyrax M55; Carl Zeiss, Jena, Germany) and heat-fixed to a microscope slide at 40˚C (Memmert 100–800; Memmert). The paraffin was removed by immersing slides in xylene substitute, twice for 10 min each. The tissue was rehydrated in a descending series of aqueous ethanol (96, 80, 70 and 60% v:v) for 10 min each and rinsed twice with deionized water for 5 min each.

The sections were stained for a minimum of 60 min with 0.005% (w:v) fluorol yellow 088 dye (Santa Cruz Biotechnology, Dallas, TX, USA) dissolved in a 1:1 (v:v) melted polyethylene glycol 4000 (SERVA Electrophoresis; Heidelberg, Germany) and 90% glycerol. The stain was washed-off with deionized water. Sections were examined by fluorescence microscopy (BX-60; Olympus Europa, Hamburg, Germany) and photographed (DP73; Olympus) under incident bright and fluorescent light (U-MWU filter, 330–385 nm excitation, ≥420 nm emission wavelength; Olympus). The number of single fruit replicates was 5.

#### Developmental time course

The developmental time courses of change in fruit mass, surface area, frequency of lenticels per unit area and the areas of lenticel cores and pores were established. The cv. ‘Apple’ mango fruits were randomly harvested from pre-selected trees (based on tree shape and flowering density) in an orchard at Mwala. Fruit mass was quantified using a balance (TX420L; Shimadzu Corporation, Kyoto, Japan). Fruit length and two orthogonal diameters (in the equatorial region) were determined using a digital caliper (CD-20PKX; Mitutoyo, Kawasaki/Kanagawa, Japan). Surface area was calculated from the above three fruit dimensions assuming sphericity. Earlier studies established that the surface area thus calculated was always within 98% of the surface area measured on excised peels [18]. A sigmoidal regression model was fitted through a plot of fruit surface area vs. time (days after full bloom; DAFB). The surface area growth rate (cm^2^ d^-1^) was calculated from the first derivative of the regression model. The number of replicates at any sampling date was 20.

Lenticels were inspected microscopically in surface view and also in cross-sections throughout fruit development. Lenticel frequency and the areas of lenticel cores and pores were quantified as above.

Lenticel width and depth were also determined microscopically using the above procedures. The relationships among the lenticel characteristics were analyzed by correlation analysis. The number of replicates was 50.

#### Lenticels in different regions of the fruit surface

The frequency of lenticels per unit surface area and the lenticel core area were quantified in the ‘stem end’, ‘cheek’, ‘apex’, ‘nak’(ventral) and ‘back’ (dorsal) regions of ‘Apple’ mango fruit as described by [19, 20]. Fruit were sampled from Mwala. See **S2 Fig** for the illustration of the different regions of the fruit surface. The ‘nak’ region contains the stylar scar and is also referred to as the ‘beak’.

#### Effect of fruit size on lenticel characteristics

The effects of size of ‘Apple’ mango fruit (site Mwala) on lenticel frequency per unit area, lenticel core area and the number of lenticels per fruit were established at maturity using fruit selected for a maximum range in size. The fruit were peeled, and the peels flattened between two glass plates. The flattened peels were photographed with a digital camera (Lumix DMC-G80; Panasonic Corporation, Osaka, Japan) fitted with a macro lens (Olympus M. Zuiko Digital 60 mm; Olympus Corporation, Tokyo, Japan). A ruler was included in each image for calibration. Peel area and lenticel frequency were quantified by image analysis (ImageJ 1.53P; National Health Institute). The number of lenticels on a whole-fruit basis was calculated as the product, lenticel frequency per unit area × fruit surface area. The number of single fruit replicates was 40.

#### Cultivar effects

Visual field observations indicated that russet susceptibility and lenticel morphology markedly differed between mango cultivars. We therefore sampled mature fruits of cvs. ‘Apple’ (171 DAFB), ‘Tommy Atkins’ (168 DAFB), ‘Ngowe’ (171 DAFB) and ‘Kitovu’ mango (204 DAFB) from a commercial orchard located in Mwala. All cultivars were subjected to the same crop husbandry and sampled at commercial maturity at the same site. Lenticel frequency and lenticel core area of the different cultivars were quantified. Fruits were rated for russet severity and photographed. Severity of russeting was quantified using a scoring scheme [11]. Russet was scored from 0 to 4 on a per fruit basis. Score 0: 0% of the fruit surface area russeted; score 1: 1–10% russeted area; score 2: 11–25% russeted area; score 3: 26–50% russeted area; and score 4: 51–100% russeted area. The number of single fruit replicates was 200 for russet severity and 40 for lenticel frequency and core area per lenticel.

#### ‘Apple’ mango from different growing sites

To assess the variability of lenticel characteristics on fruit grown at different sites, the effect of orchard was investigated. Mature ‘Apple’ mango fruits were sampled from orchards located at different altitudes, i.e., Kakuzi (altitude 1327 m), Mwala (1244 m) and Kibwezi (687 m). Fruits were rated for russet severity. Lenticel frequency and core area per lenticel were quantified. Severity of russeting was quantified using a scoring scheme [11]. Russet was scored from 0 to 4 on a per-fruit basis as described above. Daily values of rainfall, relative humidity (RH) and temperature throughout the growing season were obtained from the website of the NASA Langley Research Center (LaRC) POWER Project (NASA Langley Research Center, Hampton, VA, USA).

#### Effects of moisture exposure

The effects of surface moisture on lenticels were investigated in developing ‘Apple’ mango at the Mwala site at 33, 58, 72, 100 and 130 DAFB. The fruit surface was exposed to moisture by mounting a polyethylene Eppendorf (PE) tube to the cheek of the fruit using a non-phytotoxic silicone rubber (Dowsil™ SE 9186 clear sealant; Dow Toray Co., Tokyo, Japan) [21]. Distilled water (1 ml) was injected into the PE tube through a hole cut in the tip using a disposable syringe. The hole was then sealed with silicone rubber. The tubes were re-inspected every 2–3 days, re-filled with distilled water when necessary and re-sealed to the fruit to prevent leakage. The untreated opposite cheek of the same fruit served as control. After 8 days, the tubes were removed, fruits harvested and treated areas of skin marked using a permanent marker.

Development of microcracks at lenticels was investigated microscopically. Microcracking was indexed by quantifying the area infiltrated by the fluorescent tracer acridine orange [22]. The fruit surface was dipped in a solution of aqueous acridine orange (0.1% w/v) for 10 min. This time was sufficient for acridine orange to penetrate through any openings in the cuticle. There was no penetration through an intact cuticle. Thereafter, the surface was rinsed with distilled water and blotted dry with a soft paper towel. Lenticels were examined using a stereo microscope (MZ10F; Leica Microsystems) under incident bright light and incident fluorescent light (GFP LP filter, 480–440 nm excitation, ≥510 nm emission wavelength). Calibrated images of lenticels were taken using a digital camera (Camera DFC7000T; Leica Microsystems). The area infiltrated by acridine orange was quantified by image analysis (Image J). The number of single fruit replicates was 10.

#### Effect of lenticels on strain relaxation of the fruit skin

The effect of lenticels on the strains released following excision of an ES and subsequent isolation of the cuticular membrane (CM) and extraction of wax from the CM was investigated in mature ‘Apple’ mango fruit from Mwala [23]. The ES, both with and without lenticels, were excised from the fruit surface using a biopsy punch (8 mm diameter; Kai Europe, Solingen, Germany) and transferred to a formaldehyde-glutaraldehyde fixative solution [17]. Cuticles were isolated enzymatically by incubating the ES in 50 mM citric acid buffer containing pectinase (90 ml l^-1^; Panzym Super E flüssig; Novozymes A/S, Krogshoejvej, Bagsvaerd, Denmark), cellulase (5 ml l^-1^; Cellubrix L; Novozymes A/S) [24, 25] and 30 mM sodium azide. The pH of the solution was adjusted to 4.0 using NaOH. The solution was refreshed periodically until the cuticle separated from adhering tissue. The cuticle was rinsed (at least 5 times) with deionized water. Adhering cellular debris was removed using a soft camel-hair brush.

A square pattern of four holes was punched in the center of the cuticle using a custom-made punch. The hole pattern was needed to allow measurement of strain relaxation of an isolated and dewaxed CM even when the rim of the CM disc curled up. This was typically the case following wax extraction. The hydrated CM was spread on a glass slide and photographed under a dissecting microscope (Wild M10; Leica Microsystems; camera DP71, Olympus). The area of the CM disc (*A*_*CM*_) and the area demarcated by the four holes were quantified using image analysis (Software Cell^P, Olympus Soft Imaging Solution, Muenster, Germany).

To determine strain relaxation upon wax extraction, the dry CM disc was extracted in a soxhlet apparatus using (1:1 v:v) CHCl_3_/CH_3_OH for a minimum of 2 h. Following wax extraction, the dewaxed CM (DCM) was dried to remove any solvent residues, then rehydrated overnight using deionized water, spread on a glass slide and photographed (Wild M10; Leica Microsysteme; camera DP71, Olympus). The area demarcated by the hole pattern was re-quantified using image analysis (Software Cell^P, Olympus). The area of the entire DCM (*A*_*DCM*_) disc was then calculated from the area of the square-hole pattern.

The release of apparent strains due to excision of the ES and isolation of the CM ($\varepsilon_{exc+iso}^{\prime }$), due to wax extraction ($\varepsilon_{extr}^{\prime }$) and the sum of the two strains ($\varepsilon_{tot}^{\prime }$) were calculated using the following equations:

$$\varepsilon_{exc+iso}^{\prime }=\frac{A^{i}-A_{CM}^{i}}{A_{DCM}^{i}}\times 100$$ (1)

$$\varepsilon_{extr}^{\prime }=\frac{A_{CM}^{i}-A_{DCM}^{i}}{A_{DCM}^{i}}\times 100$$ (2)

$$\varepsilon_{tot}^{\prime }=\varepsilon_{exc+iso}^{i}+\varepsilon_{extr}^{i}$$ (3)

In these equations, *A*^*i*^ represents the area of the ES prior to excision. This area is equivalent to the cross-sectional area of the circular biopsy punch. The $A_{CM}^{i}$, represents the area of the CM disc after enzymatic isolation and the $A_{DCM}^{i}$ the area of the DCM (after wax extraction). Using these procedures, the strain relaxations of fruit-skin samples, with and without lenticels, were compared. The number of replicates was 40.

Strain relaxation of a lenticel was also quantified. For this, ES were selected having a maximum range of lenticel frequency and were excised. These were enzymatically isolated and wax extracted as described above. The core area per individual lenticel was measured before and after wax extraction. Lenticel strain ($\varepsilon_{lenticel}^{\prime }$) was quantified on lenticels within the area demarcated by the four holes using Eq 4.


$$\varepsilon_{lenticel}^{\prime }=\frac{A_{IL}^{\prime }-A_{DL}^{\prime }}{A_{DL}^{\prime }}\times 100$$ (4)

In this equation, $A_{IL}^{\prime }$ represents the core area per lenticel after isolation and $A_{DL}^{\prime }$ the core area after wax extraction.

### Data analysis

Unless otherwise stated, data are presented as means ± standard errors. Analyses of variance, regression analyses and correlation analyses were conducted using the statistical software package R (version 4.0.3; R Foundation for Statistical Computing, Vienna, Austria).

## Results

**Fig 1:**
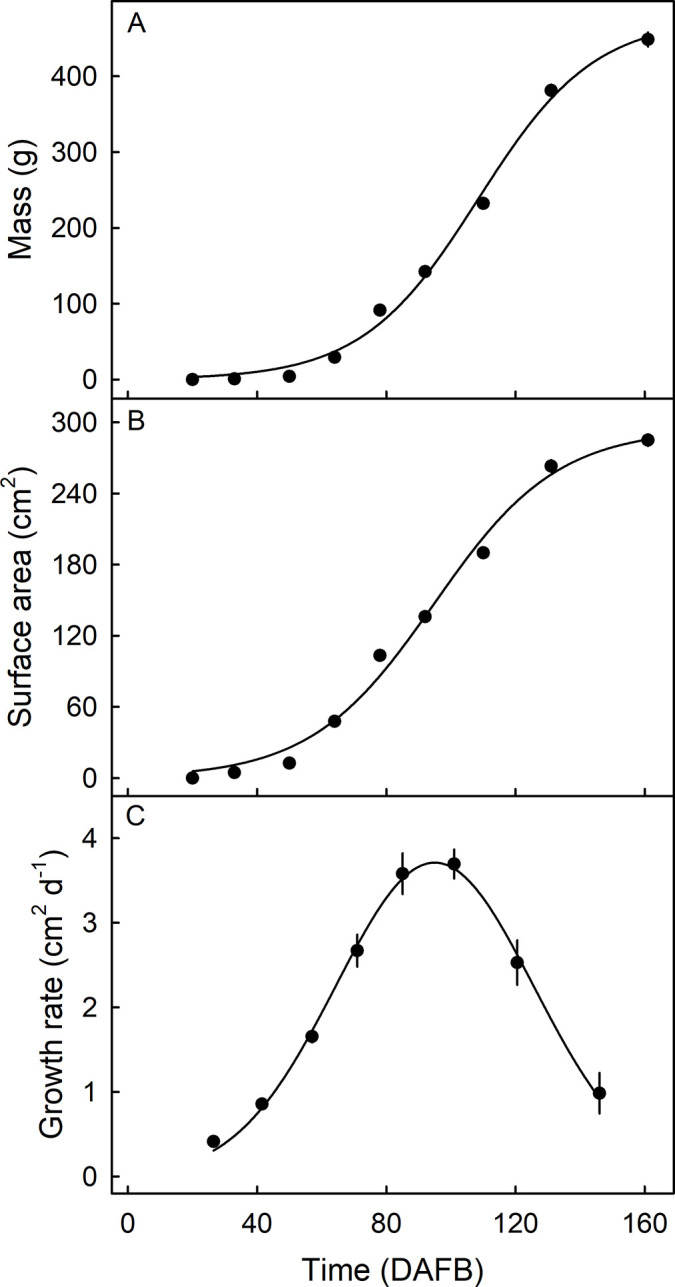
Developmental time course of change in fruit mass (A), surface area (B) and surface area growth rate (C) of ’Apple’ mango fruits grown in the same orchard. Fruit surface area was calculated from measurements of fruit length and of two orthogonal diameters in the equatorial region, and assuming sphericity. This calculation was previously validated using the following linear model: *Surface area* = 1.18×*peel area*+0.93, *r*^2^ = 0.98*** [18]. X-axis time scale in days after full bloom (DAFB). Data represent means ± standard errors. N = 20.

Fruit mass and surface area increased sigmoidally with time (Fig 1A and 1B). The surface-area growth rate reached a maximum of 3.7 cm^2^ d^-1^ at about 100 DAFB, and declined thereafter (Fig 1C).

**Fig 2:**
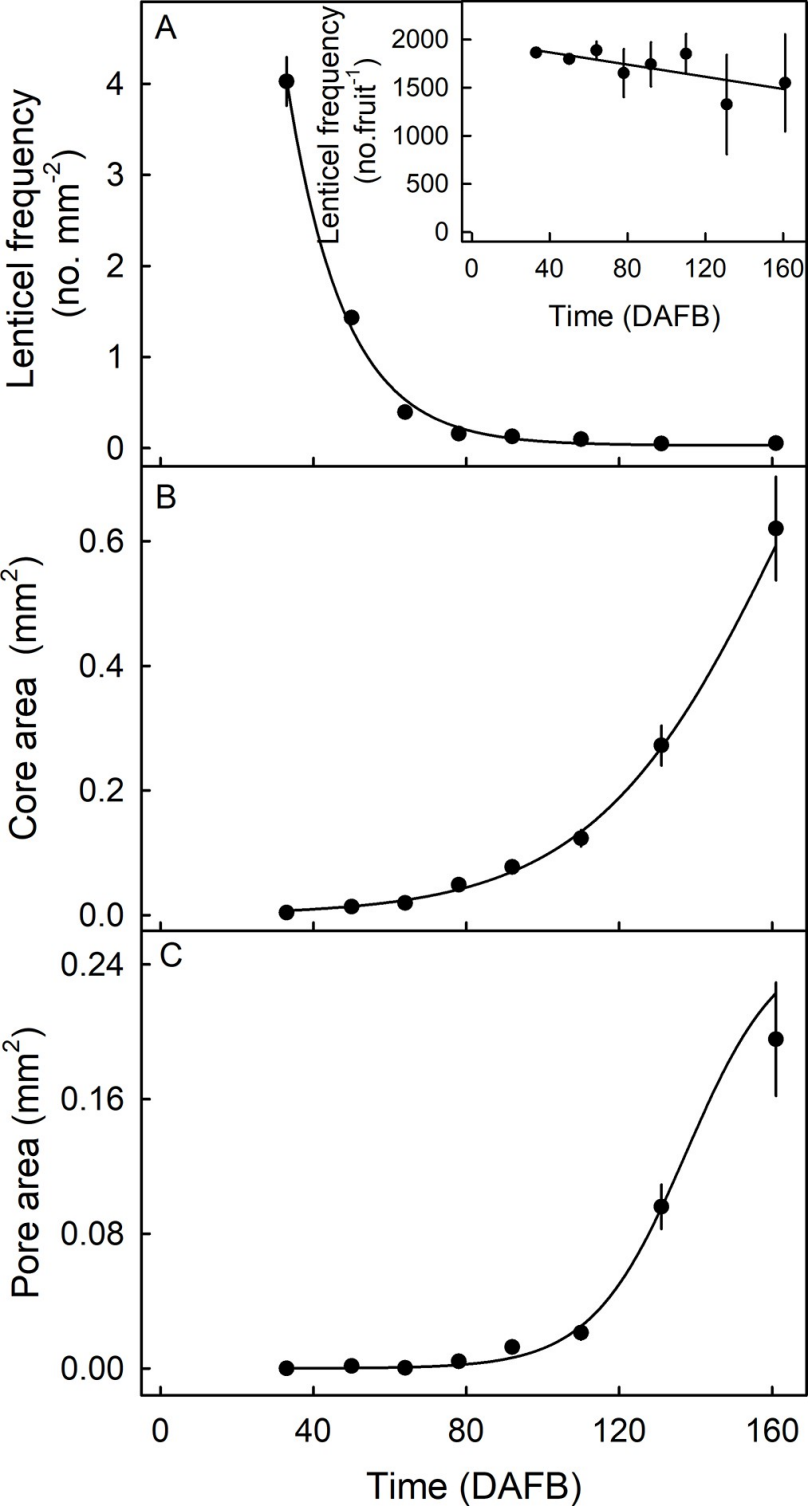
Developmental time course of changes in lenticel frequency (number per unit area and number per fruit) (A and A inset), core area per lenticel (B) and pore area per lenticel (C) in ‘Apple’ mango. The fruits were grown in the same orchard. The number of lenticels on a whole fruit was calculated as lenticel frequency per unit area × the fruit surface area. X-axis time scale in days after full bloom (DAFB). Data represent means ± standard errors. N = 30.

The frequency of lenticels per unit surface area decreased during development, whereas the lenticel core area and pore area increased (Fig 2A–2C). The number of lenticels per fruit remained essentially constant (Fig 2A, inset).

**Fig 3:**
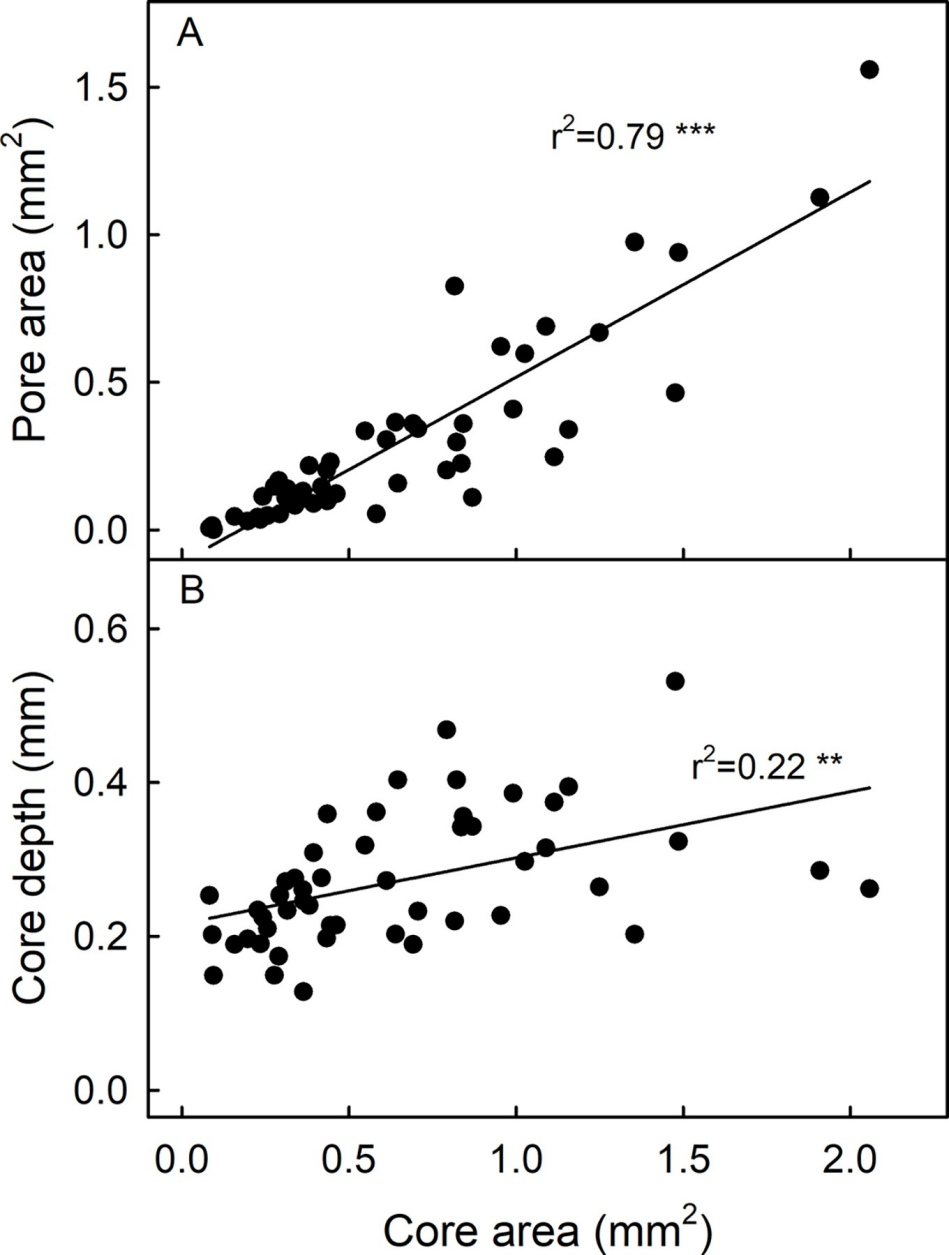
Correlation between lenticel pore area and lenticel core area (A) and lenticel core depth and lenticel core area (B) on mature ‘Apple’ mango. The fruits were grown in the same orchard. Lenticel morphology and cross-section were examined microscopically. N = 50.

Lenticel core area and pore area were significantly and positively correlated (r^2^ = 0.79 ***). In contrast, core depth had only a weak relationship with core area (r^2^ = 0.22 **) (Fig 3A and 3B).

The distribution of lenticels over the fruit surface was not uniform (Table 1). Lenticels were most frequent in the distal region and least in the cheek and the nak regions. The cheek region also had lenticels with the largest core areas, whereas the core areas of those in the apex region were significantly smaller (Table 1).

**Table 1:** Peak growth rate, lenticel frequency and area per lenticel in different regions of mature ‘Apple ‘mango (See S2 Fig for nomenclature). The fruit were grown in the same orchard. Data is represented as means ± standard errors. N = 40.

Region	Peek growth rate (cm^2^ d^-1^)	Lenticels	
		Frequency (No. mm^-2^)	Core area (mm^2^)
**Stem end**	0.05 ± 0.01^a^	0.07 ± 0.00 b^b^	0.18 ± 0.02 b
**Cheek**	0.07 ± 0.02	0.03 ± 0.00 a	0.46 ± 0.05 c
**Apex**	0.06 ± 0.02	0.09 ± 0.03 c	0.04 ± 0.00 a
**Back**	0.07 ± 0.02	0.06 ± 0.00 b	0.16 ± 0.02 b
**Nak**	0.07 ± 0.02	0.04 ± 0.00 a	0.16 ± 0.02 b

^a^Peek growth rate data is taken from [18].

^b^Mean separation within columns by Tukey studentized range test, P≤ 0.05.

**Fig 4:**
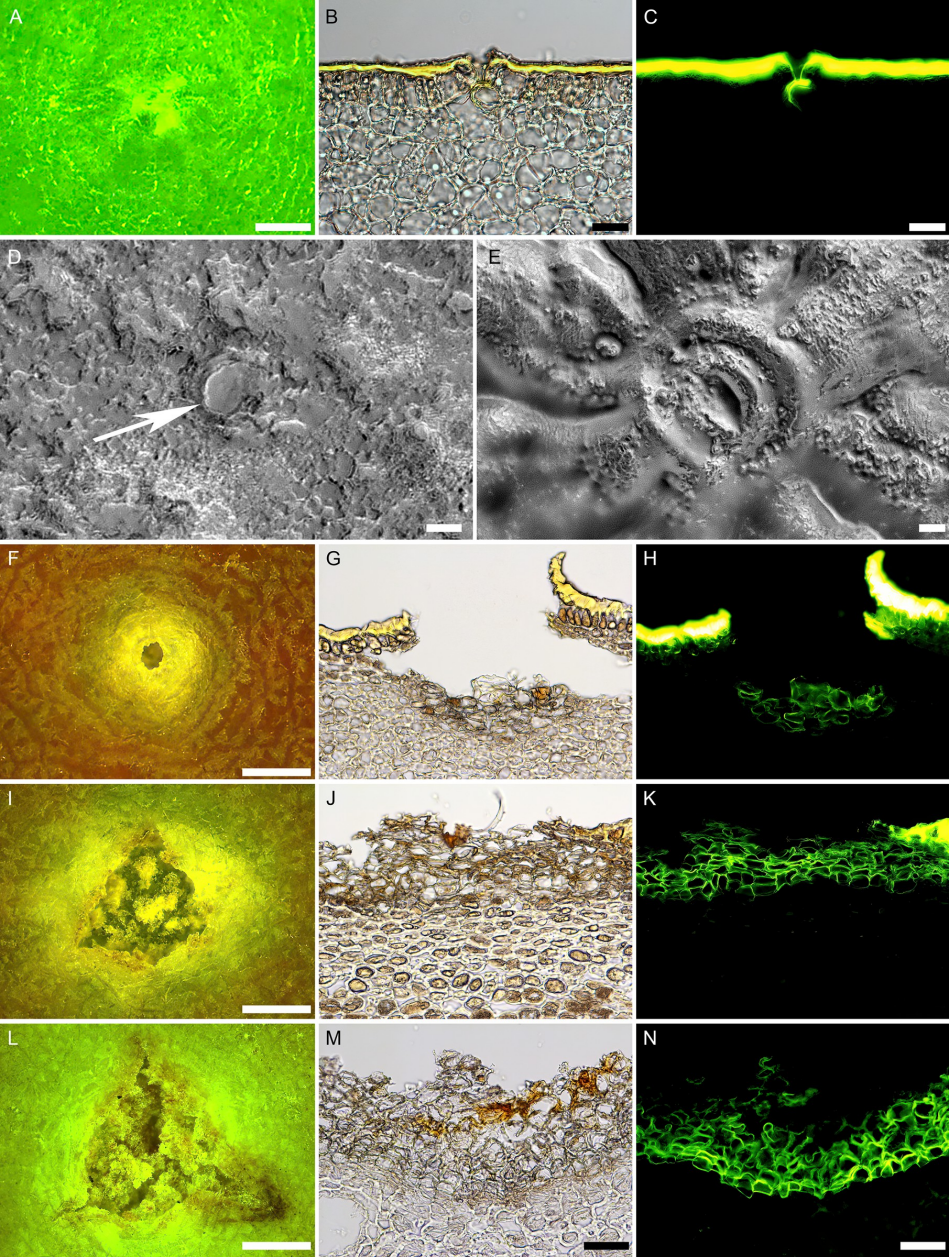
Surface (left) and cross-sectional views (middle and right) of stomata and lenticels in developing ’Apple’ mango fruits from the same orchard. Fruits were sampled at 33 (A-E), 78 (F-H), 110 (I-K) and 161 (L-N) days after full bloom (DAFB). Surface view was examined under a binocular microscope and a digital microscope. Cross-sections were examined microscopically under incident (middle) and fluorescence (right) light. White arrow indicates stomal pores plugged with wax. Scale bars are 250 (A), 20 (B,C), 10 (D,E), 500 (F, I, L) and 50 μm (G,H,J,K,M,N). N = 10 (surface views) and 5 (cross-sections).

Microscopy at 33 and 78 DAFB revealed stomata on the surface that appeared as whitish spots with no visible pore (Fig 4A). Heavy wax deposits covered the guard cells and stomatal pores completely at 33 DAFB (Fig 4D). The stomatal apparatus became clearly visible only after removal of epicuticular waxes (Fig 4E). Staining with fluorol yellow revealed a cuticle above the epidermal cells. At the guard cells, the cuticle extended into the stomatal antechamber (Fig 4B and 4C). By 78 DAFB, stomata appeared as large openings, the rim of the cuticle surrounding the pore was still intact. Cross-sections revealed the onset of periderm formation several cell layers below the pore (Fig 4F–4H). At 110 DAFB, cracks in the cuticle had begun to form that tore the rim of the pore. By 161 DAFB, the pore rim was torn, and severe cracks developed in the cuticle. The periderm extended laterally, the number of phellem layers increased and eventually filled the entire core volume of the lenticel (Fig 4I–4N).

Compared to a lenticel-free ES, the presence of lenticels increased the strain released by between 1.5- and 1.8-times when an ES was excised and the subsequent isolation of the cuticle $\left(\varepsilon_{exc+iso}^{\prime }\right)$ and the extraction of cuticular wax $\left(\varepsilon_{extr}^{\prime }\right)$. The sum of these strains, i.e., the total strain, was 1.7 times larger for cuticles with lenticels as compared to those without lenticels (Table 2).

**Table 2:** Effect of the presence of lenticels on apparent strains (%) in ‘Apple’ mango skins from the same orchard. Apparent strain was partitioned into strains due to excision and isolation ($\varepsilon_{exc+iso}^{\prime }$) and wax extraction ($\varepsilon_{extr}^{\prime }$). The latter two strains were summed up to make the total strain ($\varepsilon_{tot}^{\prime }$). Data is represented as means ± standard errors. N = 30.

Treatment	$\varepsilon_{exc+iso}^{\prime }$	$\varepsilon_{extr}^{\prime }$	$\varepsilon_{tot}^{\prime }$
**With lenticels**	12.6 ± 0.6 b^a^	28.1 ± 1.0 b	40.7 ± 1.0 b
**Without lenticels**	8.5 ± 0.6 a	15.8 ± 0.7 a	24.4 ± 0.9 a

^a^Mean separation within columns by Tukey studentized range test, P≤ 0.05

**Fig 5:**
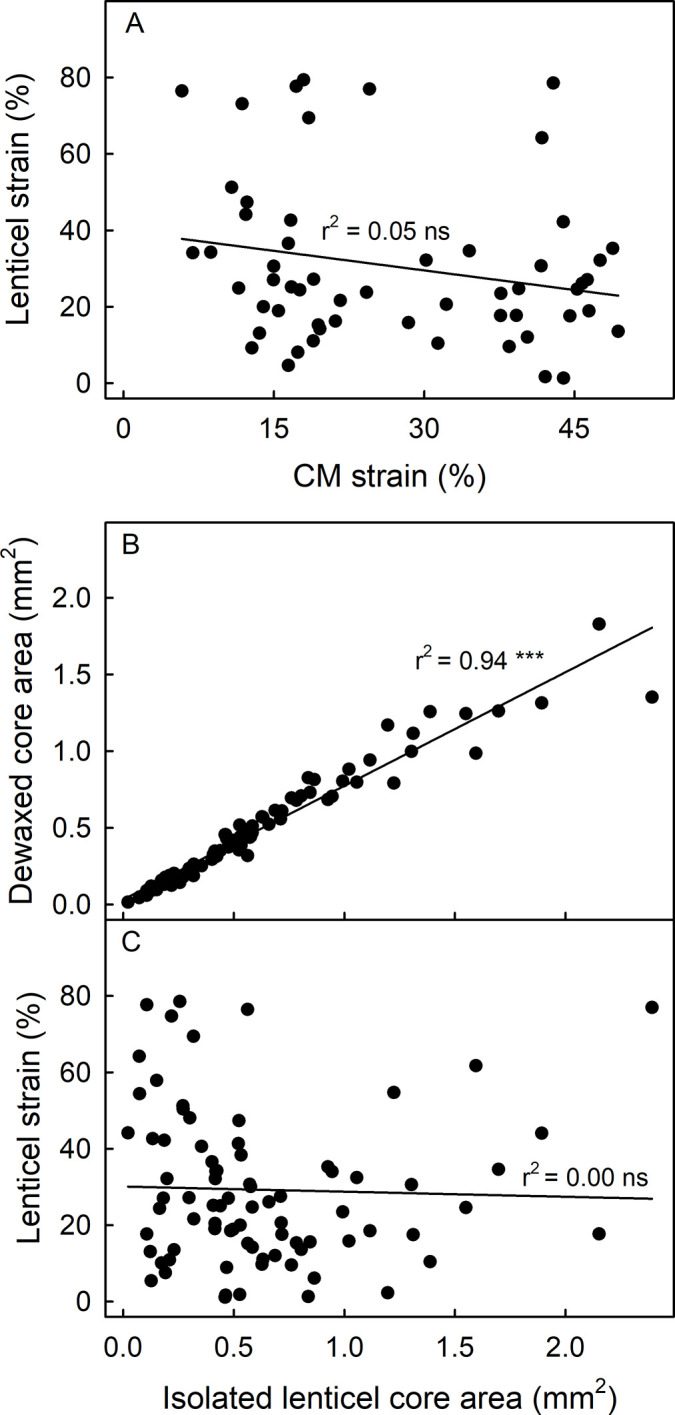
Correlation between the strain released from a lenticel and that released from an excised and isolated cuticular membrane (CM) (A), between the dewaxed core area of a lenticel and the core area in the isolated CM disc (B), and between the strain released from a lenticel and the core area in the isolated CM disc (C). Lenticel core areas in isolated cuticles and in dewaxed cuticles were analyzed by image analysis. N = 40 (lenticels) and 24 (cuticles). The slope of the regression line in B is 0.74 ± 0.02.

There was no relationship between the strain released from a lenticel and that from the corresponding isolated cuticular membrane (Fig 5A). However, the area of the lenticel core following wax extraction was closely related to the area of the lenticel core in the isolated cuticle disc (Fig 5B). The strain released from a lenticel upon isolation and upon wax extraction was highly variable and not related to the core area in the isolated cuticle disc (Fig 5C).

**Fig 6:**
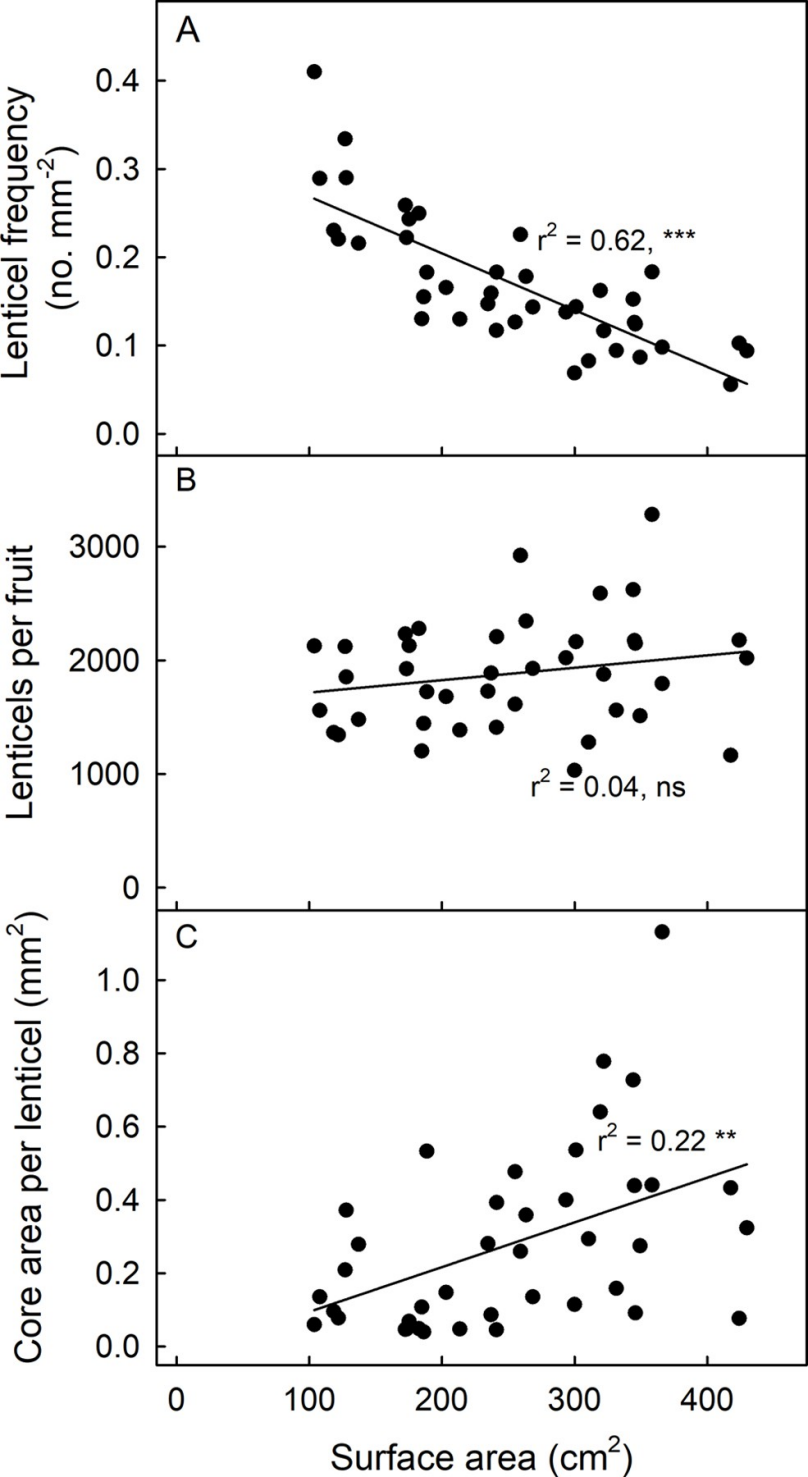
Effect of fruit surface area on frequency of lenticels per unit area (A), total lenticels per fruit (B) and the core area per lenticel (C) of mature ‘Apple’ mango fruit grown under same conditions. The fruit surface area was measured by peeling the fruit, flattening the peel between two glass plates, photography and finally by image analysis. N = 40. For regression equations see the S1 File.

The number of lenticels per unit area of fruit surface was negatively related with the surface area of the fruit (r^2^ = 0.62 **) (Fig 6A). On a whole-fruit basis, there was no significant difference in the numbers of lenticels between small and large fruit (Fig 6B). The core area per lenticel and the fruit surface area were weakly and positively related (r^2^ = 0.22**) (Fig 6C).

**Fig 7:**
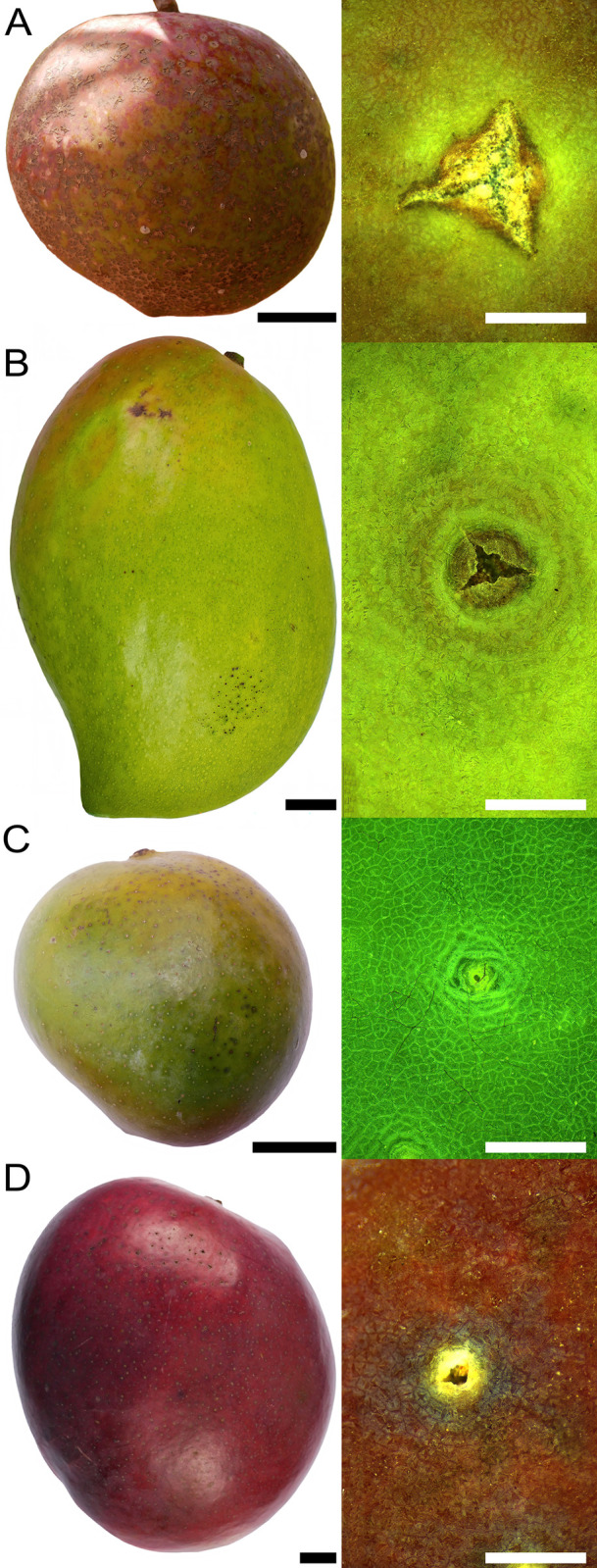
Macroscopic view of mature ’Apple’ (A), ’Ngowe’ (B), ’Kitovu’ (C) and ’Tommy Atkins’ (D) fruit and microscopic view of lenticels. All fruit were grown at the same site in the same season. Scale bars are 2 cm for macrographs (A-D) and 1 mm for micrographs.

Compared to ‘Ngowe’, ‘Kitovu’ and ‘Tommy Atkins’ mangos, ‘Apple’ had the lowest frequency of lenticels and the largest core area per lenticel. Furthermore, russeting was most severe in ‘Apple’ (Table 3, Fig 7).

**Table 3:** Severity of russet, frequency and areas of lenticels in different mango cultivars. All cultivars were sampled from the same orchard. Severity of russet was quantified using a rating scale from 0 to 4: score 0: 0% of the fruit surface area russeted; score 1: 1–10% russeted area; score 2: 11–25% russeted area; score 3: 26–50% russeted area; and score 4: 51–100% russeted area. Lenticel properties were quantified on the cheek of the fruit. Data is represented as means ± standard errors. N = 200 (russet severity) and 40 (lenticel frequency and area per lenticel).

Cultivar	Russeting (Score)	Lenticels	
		**Frequency (No. mm** ^**-2**^ **)**	**Core area (mm** ^**2**^ **)**
**Apple**	1.15 ± 0.05 b^a^	0.05 ± 0.00 a	0.50 ± 0.04 c
**Ngowe**	0.03 ± 0.01 a	0.19 ± 0.01 c	0.16 ± 0.01 b
**Kitovu**	0.00 ± 0.00 a	0.15 ± 0.01 b	0.03 ± 0.00 a
**Tommy Atkins**	0.01 ± 0.01 a	0.33 ± 0.02 d	0.05 ± 0.01 a

^a^Mean separation within columns by Tukey studentized range test, P≤ 0.05.

Considerable differences in lenticel frequency and core area per lenticel were observed between different growing sites of ‘Apple’ mango. Russeting was most severe in Kakuzi, intermediate in Mwala and almost absent in Kibwezi. Fruit from Kakuzi exhibited the largest lenticels and the lowest lenticel frequency. On the other hand, fruit from Kibwezi had the smallest lenticels and the highest lenticel frequency. It is interesting that fruit from the three sites also differed in (1) russeting and (2) growing conditions, as indexed by altitude, rainfall and temperature which differed markedly between the three sites. Summarizing, the russeted Kakuzi fruit grew at high altitude and were exposed to high rainfall and low temperatures. In contrast, the non-russeted Kibwezi fruit grew at lower altitude and was exposed to lower rainfall and higher temperatures. Intermediate-russeting was observed on fruit from Mwala which is at intermediate altitude, and experiences intermediate rainfall and temperature (Table 4).

**Table 4:** Effect of orchard location on russeting and lenticel frequency and lenticel area in cv. ‘Apple’ mango. The sites were selected based on their differences in altitude—meters above sea level (m.a.s.l.). The climatic variables include: cumulative rainfall, relative humidity (RH), minimum and maximum temperatures. Severity of russet was quantified on a 0 to 4 scoring scheme: Score 0: 0% of the fruit surface area russeted; score 1: 1–10% russeted area; score 2: 11–25% russeted area; score 3: 26–50% russeted area; and score 4: 51–100% russeted area. Data is presented as means ± standard errors. N = 200 (Russeting score) and 40 (lenticel frequency and lenticel area).

Orchard Location	Altitude (m.a.s.l.)	Cumulative rainfall (mm)	RH (%)	Temperature (°C)		Russeting (score)	Lenticels	
				Min.	Max.		Frequency (No. mm^-2^)	Core area (mm^2^)
**Kibwezi**	687	125.7	64.0 ± 0.5	17.1 ± 0.1	30.9 ± 0.2	0.03 ± 0.01 a	0.14 ± 0.01 b	0.03 ± 0.00 a
**Mwala**	1244	396.0	68.6 ± 0.8	15.4 ± 0.1	28.2 ± 0.2	1.15 ± 0.05 b	0.05 ± 0.00 a	0.50 ± 0.04 b
**Kakuzi**	1327	459.5	67.4 ± 0.8	16.5 ± 0.1	29.0 ± 0.2	1.72 ± 0.10 c	0.04 ± 0.00 a	1.08 ± 0.08 c

Mean separation by Tukey studentized range test, P≤ 0.05. Means within a column followed by the same letter are not significantly different.

**Fig 8:**
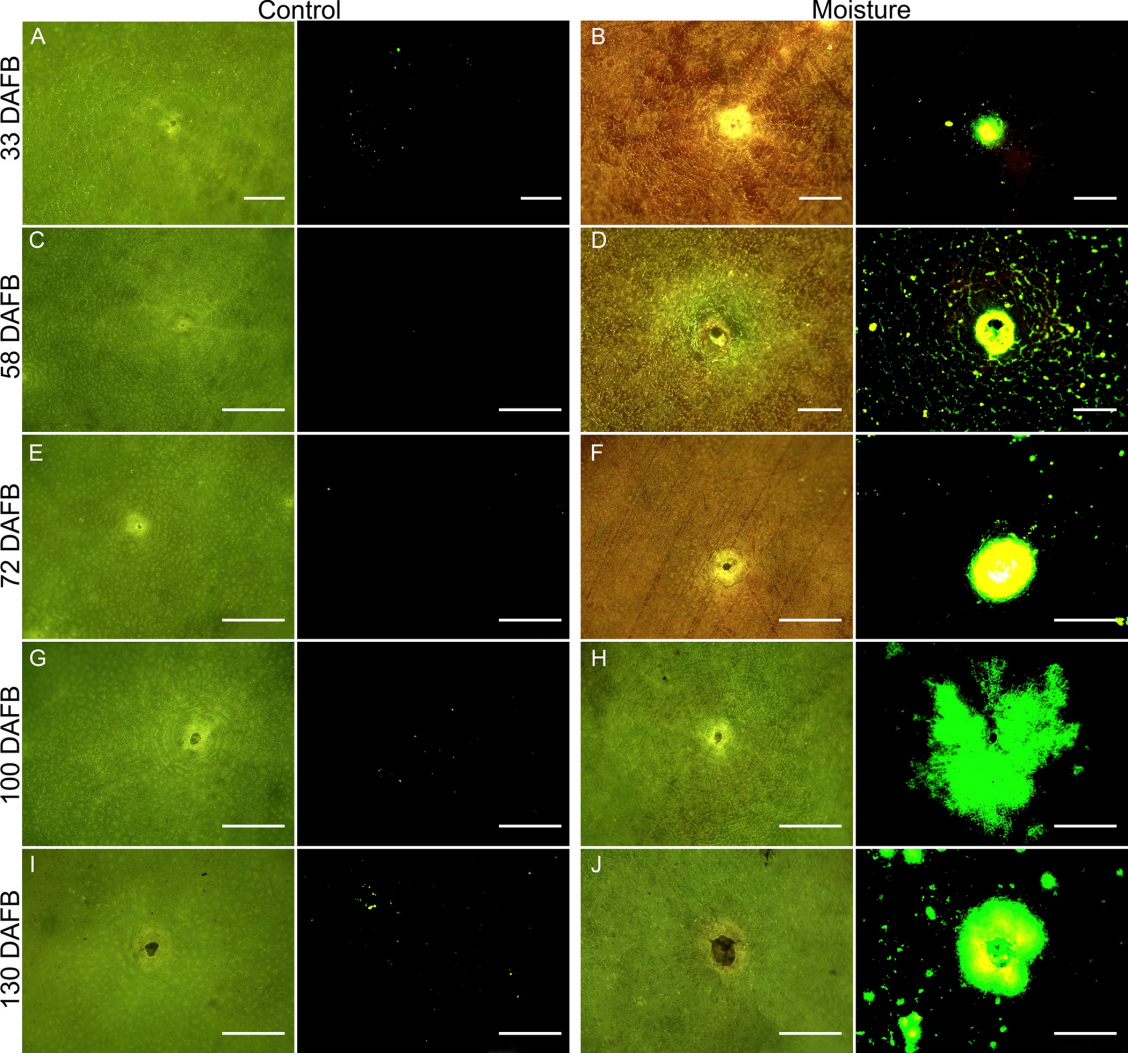
Micrographs of lenticels of developing ‘Apple’ mango sampled 33 (A-B), 58 (C-D), 72 (E-F), 100 (G-H) and 130 (I-J) days after full bloom (DAFB). The fruit surfaces were either left untreated (A, C, E, G, I) or exposed to surface moisture (B, D, F, H, J). Moisture was presented to a small region on the cheek of a fruit for 8 days by attaching an Eppendorf tube containing water. The surface was later viewed under bright or fluorescence light. N = 10. Scale bar is 500 μm.

**Fig 9:**
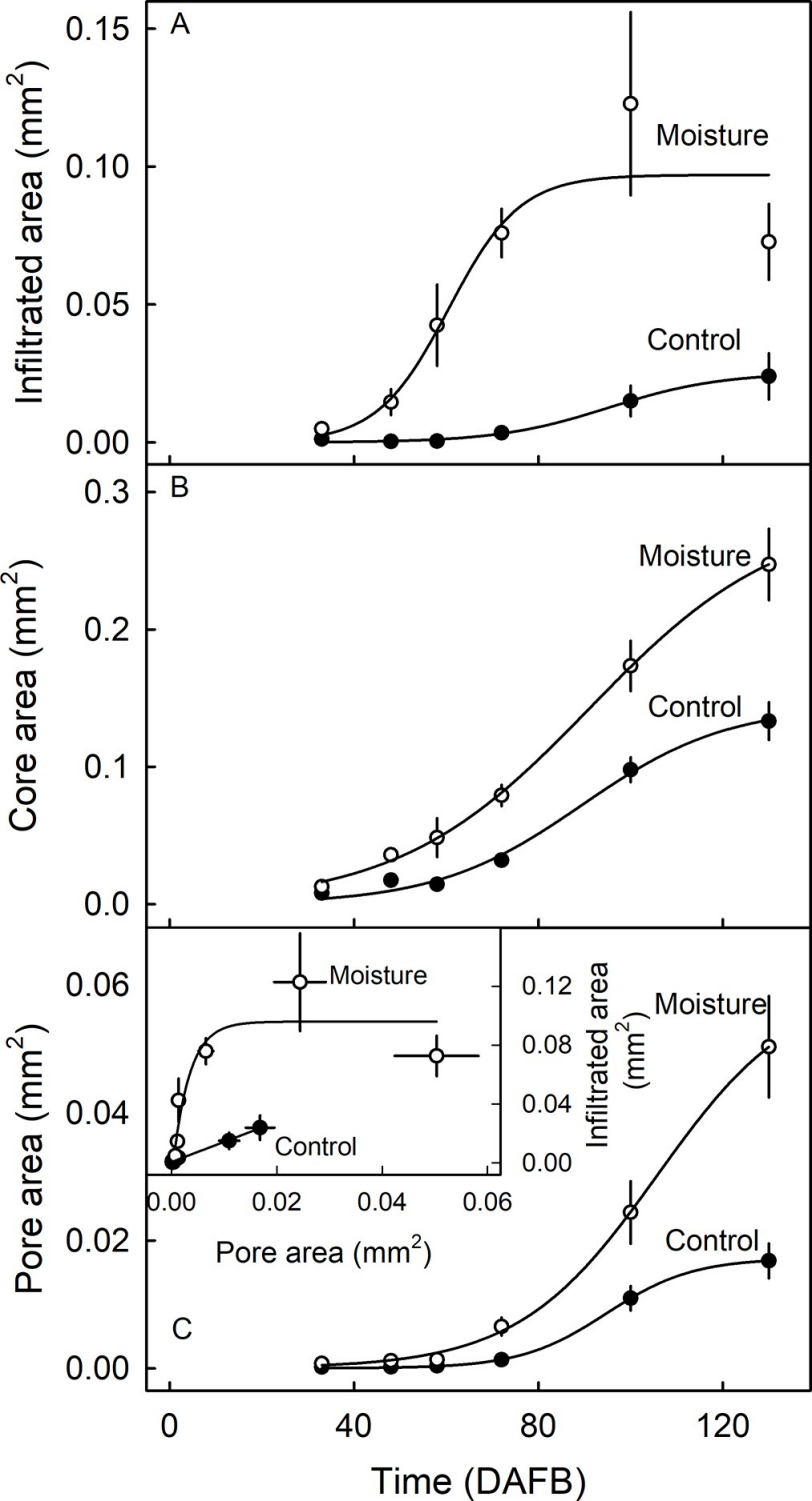
Effect of moisture exposure of the fruit surface of developing ‘Apple’ mango on (A) the area surrounding a lenticel that is infiltrated by the fluorescent tracer acridine orange, (B) the lenticel’s core area and (C) the lenticel’s pore area. Inset in C: Infiltrated area as affected by the pore area of the lenticel. Unexposed fruit served as controls. X-axis scale is in days after full bloom (DAFB). The number of replicates ranged from 19 to 46.

On the same fruit, lenticels exposed to moisture fluoresced more than unexposed lenticels (Fig 8). The fluorescing area around lenticels and their core and pore area were small during early development, then increased towards a maximum around 100 DAFB (Figs 8 and 9). The core area, the pore area and the infiltrated area per lenticel in moisture-exposed fruit were consistently larger throughout development than in unexposed control fruit (Fig 9).

## Discussion

This discussion focusses on the following three findings;

- growth strains enlarge lenticels and lead to their eventual rupture,
- moisture on the fruit surface increases russet formation by increasing lenticel cracking and
- lenticels are sites of russet initiation in ‘Apple’ mango.

### Growth strain enlarges lenticels leading to their rupture

Lenticels on ‘Apple’ mango increase in both core and pore area during development and this leads to their eventual rupture. The driver for lenticel enlargement and lenticel rupture is the progressive increase in growth strain in the fruit skin [26]. This hypothesis is supported by the following arguments.

First, there was a positive correlation between fruit surface area and lenticel core area (Fig 4). Although the number of lenticels per unit area decreased during development, the number of lenticels per fruit basis did not change–this indicates new lenticels did not appear during fruit development, instead, as the fruit expanded, a constant number of lenticels was distributed over an increasing surface area. The increases in core and pore areas are consistent with this observation. The increase in pore area of a lenticel was simply related to the expansion of the core area. By about 110 DAFB, the limits of extensibility of the lenticels were exceeded and the lenticels started to rupture (Fig 3). ‘Apple’ mango is not unique in this behavior [14, 27, 28].

Second, the lenticel core area differed between regions on a fruit and these differences were simply related to the rates of fruit surface-area expansion in these regions [18]. We observed larger core areas and a lower frequencies of lenticels in the cheek region, where the rates of increase in surface area are largest [18]. In contrast, the apex region of the fruit, where the rates of surface area growth are low, lenticel frequency was high and the lenticel core area was small [18]. These findings are consistent with earlier reports for *Malus* apple (*Malus × domestica* Borkh.) [4]. In *Malus* apple, lenticel core area decreases and lenticel frequency increases from the pedicel end of the fruit to the calyx end [4], whereas the surface growth rate decreases [29].

Third, the time of lenticel rupture and of crack extension into the adjacent cuticle and epidermis, coincides with the time of maximum surface area expansion rate. This suggests a cause (surface growth strain) and effect (cracking of lenticels and surrounding cuticle) relationship. Apparently, the increase in core area of the lenticels was not sufficient to accommodate the increase in fruit surface area that accompanies the growth of the underlying tissues [8]; this results in the cracking of the cuticle, immediately adjacent to the lenticel. It also consistent with the higher strain relaxation of skin segments containing lenticels, compared to skin segments with no lenticels.

Fourth, lenticels developed from stomata. At 33 DAFB, we observed stomata but no lenticels. By 78 DAFB, the stomata were gaping and an upward bending of the cuticle surrounding the stomatal pore indicated severe tangential strain. By 110 DAFB, lenticels were fully developed, as indexed by the presence of a fully developed periderm. Similarly, in ‘Kensington Pride’ and ‘Namdokmai’ mango cultivars, stomata ruptured and developed into lenticels [13, 30]. In ‘Namdokmai’ mango, lenticels were fully developed one month after full bloom [13]. Lenticels also develop from ruptured stomata in *Malus* apples [27].

These arguments demonstrate that in ‘Apple’ mango growth strain is causal in the development of lenticels and their cracking. Interestingly, we observed no cracking of lenticels in ‘Tommy Atkins’ or ‘Ngowe’. Both ‘Tommy Atkins’ and ‘Ngowe’ typically have larger fruit, so implying even higher skin strain than in the smaller ‘Apple’ mango–yet there was no sign of cracking [18]. This observation demonstrates that lenticel morphology also depends on cultivar [9, 10, 31].

### Surface moisture induces lenticel cracking

Growth strain is not the only factor involved in lenticel development in ‘Apple’ mango. Our study provides direct evidence that surface moisture plays a critical role in lenticel development, expansion and cracking.

First, the core and pore areas of lenticels exposed to moisture were as much as 2-fold larger than those of control lenticels unexposed to water.

Second, the area infiltrated by acridine orange was larger for moisture exposed lenticels then for un-exposed lenticels, on the same fruit. Even for lenticels having the same core and pore areas, lenticels exposed to moisture had significantly larger areas of fluorescence around them, than unexposed lenticels. The increase in fluorescing area is explained by increased microcracking around and through the lenticels. Microcracks serve as pathways through the cuticle for rapid dye uptake.

Third, the effects of moisture on lenticel core area and pore area also account for the differences in lenticels between mangos grown at different sites. Fruits grown at lower altitudes (Kibwezi) developed smaller lenticel cores than those at higher altitudes (Kakuzi). Kibwezi has a hot, dry climate with low rainfall, while Kakuzi is cooler, more humid and with higher rainfall. Consequently, fruits grown at Kakuzi had larger lenticels and were more russeted than fruits grown in Kibwezi. These orchard observations are consistent with the effects of moisture exposure on lenticel development and russeting reported in this and in our previous study [11].

A likely explanation for increased rupture of lenticels and microcracking of the cuticle around lenticels is the effect of hydration on the mechanical properties of the cuticle. It is well established that hydrated cuticles fracture at lower forces than dry cuticles [32–34]. In addition, lenticels function as stress concentrators. They represent islands of differing extensibility that would serve to focus growth stresses at these locations on the fruit surface [35, 36]. The lenticels in ‘Apple’ mango, would appear to be weak spots, since star-like cuticular microcracks radiate from the gaping lenticels. This was also reported in our earlier study [11]. Our earlier study, found that when cuticles were subjected to uniaxial tensile tests, cuticular failure occurred most frequently across or around the lenticels [18]. In *Malus* apple, a periderm similarly forms under ruptured stomata or in response to microcracking, due to surface moisture [21, 37–39].

### Cracking of lenticels triggers russeting in ‘Apple’ mango

In ‘Apple’ mango, almost all cuticular microcracking develops around lenticels. Russeting in ‘Apple’ mango begins at lenticels and then gradually spreads over the fruit surface [11]. In botanical terms, russeting represents the formation of a periderm, comprising a cork cambium or phellogen that divides to produce stacks of cork cells, the so called phellem cells [2, 40]. A periderm forms as a typical wound response [26]. Russeting in ‘Apple’ mango would seem to be similar to that in *Malus* apple [40]. In both cases, a periderm forms in response to a wound, i.e., a cracked lenticel in ‘Apple’ mango [11] or a microcrack in the cuticle of *Malus* apple [41]. As in *Malus* apple, the periderm forms in the hypodermal cell layers just below the stomata, as indexed by the staining of the suberized cell walls of the phellem [21, 38]. As development progresses, the thickness of the phellem and hence the depth of the lenticels increases [4]. When the fruit’s primary surface (the epidermis and the remains of the cuticle) is sloughed off, the phellem and hence the brown lenticels are clearly visible at the surface. This understanding applies to both ‘Apple’ mango and to *Malus* apple [4, 11, 16, 38]. The above arguments indicate that the initiation and development of russeting in ‘Apple’ mango follows an essentially identical path to that found in *Malus* apple.

## Conclusion

The results demonstrate that russeting in ‘Apple’ mango is initiated at lenticels. Moisture exposure of developing fruit results in a weakening of lenticels and hence the formation of strain cracking at the lenticels. The wounds and associated increases in water loss, are healed and a degree of waterproofness regained, by the formation of a subtending periderm. The lenticels of ‘Apple’ mango are much larger than those of the other mango cultivars examined. In addition to this genotypic effect, exposure of developing ‘Apple’ mango fruits to moisture results in cracking and further increases in lenticel size. It is not clear why moisture should have this effect. The relative weakness of these small areas of periderm probably results from one or several of the following: (1) the large intercellular air-spaces within the periderm of a lenticel and/or (2) the infiltration of lenticels by surface moisture and the subsequent hydration of the periderm cell walls. Coupled with this, the periderm cell walls may have low wax content or their suberization may be incomplete. All these factors will likely reduce cell:cell adhesion within the periderm.

From a horticultural point of view, it is reasonable to conclude that any reduction of exposure of the skins of ‘Apple’ mangos to moisture is likely to reduce both lenticel cracking and hence russeting. Obvious strategies to achieve this will include focusing the production of ‘Apple’ mango on sites that enjoy drier climates. Alternative strategies to reduce surface moisture should include bagging the developing fruits on the tree [42], and another strategy would be to cultivate the ‘Apple’ mangos trees under a rain shelter, on a dwarfing rootstock. These avenues for mitigation of russeting in ‘Apple’ mango all merit study.

## Supporting information

S1 Fig Fruit skin illustrating the lenticular core (red cycle) and pore (blue circle). We refer to the lenticel pore as the opening and the lenticel core as the area of loosely packed complementary cells including those subtending the pore.
(TIF)

S2 Fig Sketch of mango fruit illustrating the different regions of the fruit surface (Stem end, cheek, apex, back and nak) where skin segments were sampled. (TIF)

S1 File This is the excel file containing the data in Figs 1–3, 5, 6 and 9 and Tables 1–4. (XLSX)
