# Supplementary figures and images for: Lenticels are sites of initiation of microcracking and russeting in ‘Apple’ mango
Source: PLoS One. 2023 Sep 1;18(9):e0291129. doi: 10.1371/journal.pone.0291129 (PMC10473472; doi:10.1371/journal.pone.0291129)

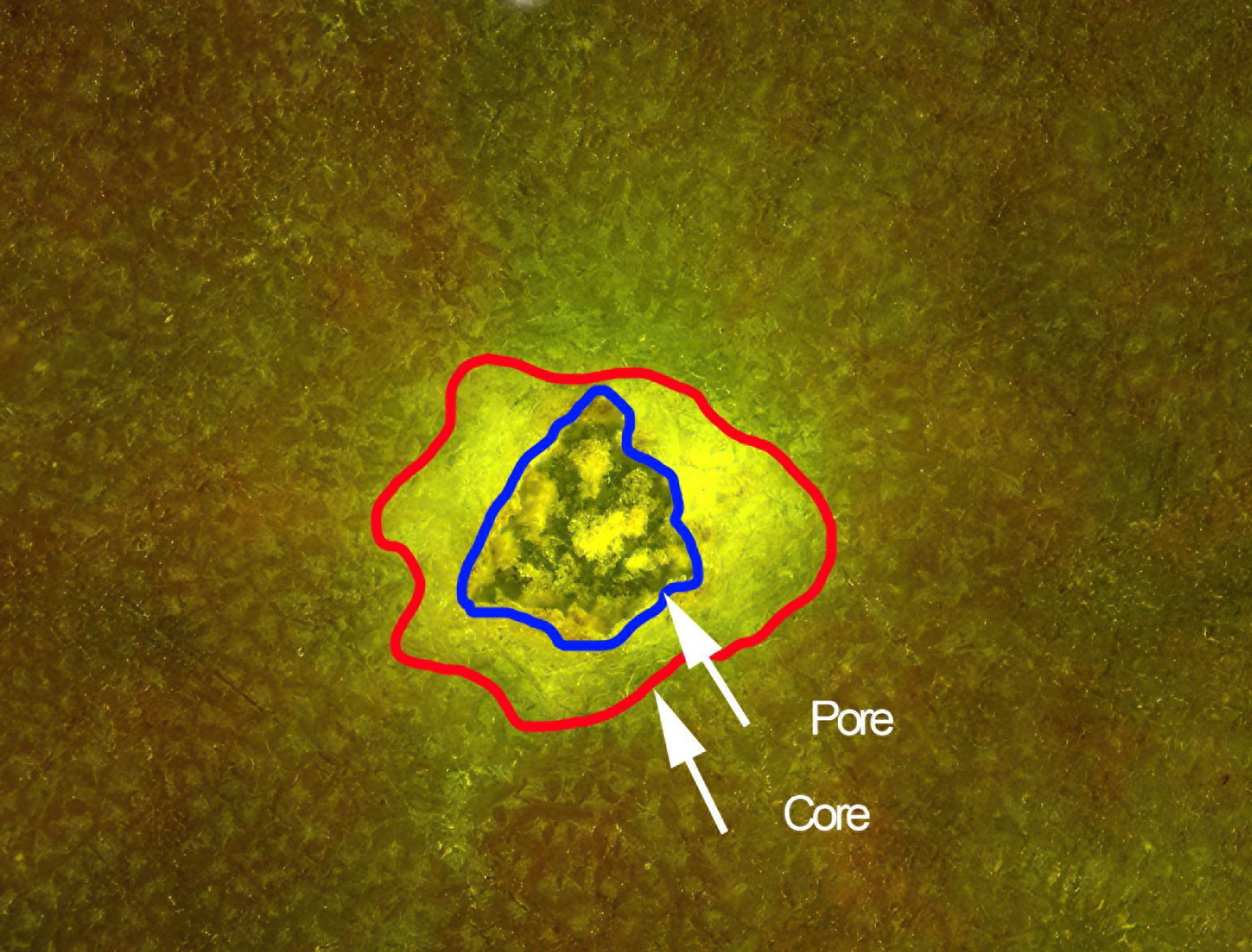

Supplement: S1 Fig — We refer to the lenticel pore as the opening and the lenticel core as the area of loosely packed complementary cells including those subtending the pore. (TIF) [file pone.0291129.s001.tif]

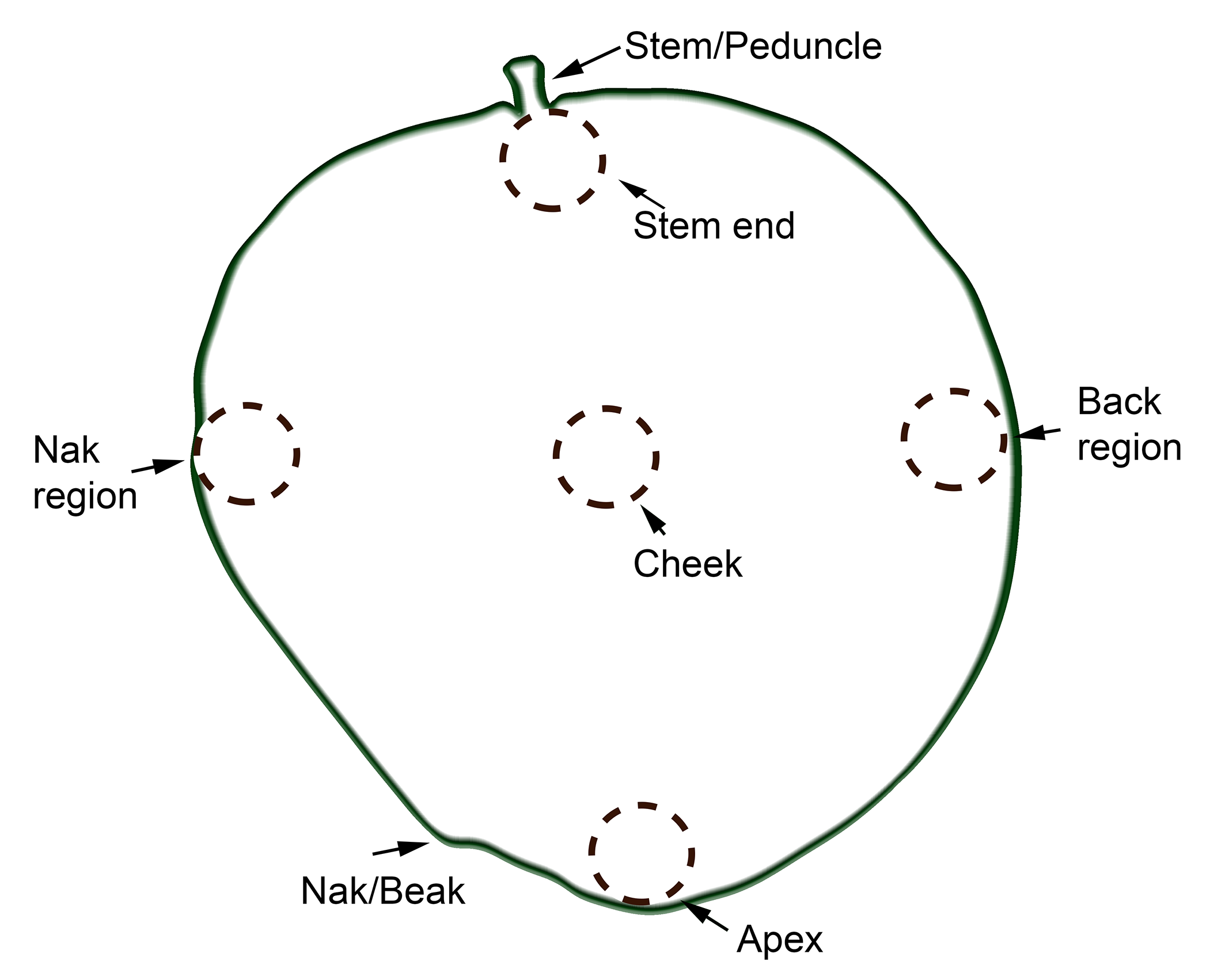

Supplement: S2 Fig — (TIF) [file pone.0291129.s002.tif]
